# Requirements for Large Scale Web Accessibility Evaluation

**DOI:** 10.1007/978-3-030-58796-3_33

**Published:** 2020-08-10

**Authors:** Fabio Paternò, Francesca Pulina, Carmen Santoro, Henrike Gappa, Yehya Mohamad

**Affiliations:** 8 Institute Integriert Studieren, JKU Linz, Linz, Austria; 9 Jack Baskin School of Engineering, UC Santa Cruz, Santa Cruz, CA USA; 10 Dipartimento di Meccanica, Politecnico di Milano, Milan, Italy; 11 Support Centre for Students with Special Needs, Masaryk University Brno, Brno, Czech Republic; 12 CNR-ISTI, Human Interfaces in Information Systems Laboratory, Via Moruzzi 1, Pisa, Italy; 13 Fraunhofer-Institute for Applied Information Technology, Sankt Augustin, Germany

**Keywords:** Accessibility guidelines, Automatic evaluation, Monitoring

## Abstract

The recent European legislation emphasizes the importance of enabling people with disabilities to have access to online information and services of public sector bodies. To this regard, automatic evaluation and monitoring of Web accessibility can play a key role for various stakeholders involved in creating and maintaining over time accessible products. In this paper we present the results of elicitation activities that we carried out in a European project to collect experience and feedback from Web commissioners, developers and content authors of websites and web applications. The purpose was to understand their current practices in addressing accessibility issues, identify the barriers they encounter when exploiting automatic support in ensuring the accessibility of Web resources, and receive indications about what functionalities they would like to exploit in order to better manage accessibility evaluation and monitoring.

## Introduction

Web accessibility is evolving because it has to respond to the demands posed by the digital transformation of our society while considering the changes in the interactive technologies used for implementing and accessing Web sites. Such changes have stimulated also new developments in terms of the associated accessibility guidelines standards, and new obligations imposed on public sector bodies as regards the compliance of public Web services. The recent WAD directive aims to obtain large scale monitoring of the adoption of accessibility guidelines. For this reason, all people involved in the commissioning and development of Websites and mobile applications of public sector bodies need to act towards providing Web services accessible for all citizens. To this end, they also need to be supported in the process of evaluating and monitoring the accessibility of their services. Thus, in the course of the EU-funded research project WADcher^1^ (Web Accessibility Directive Decision Support Environment) we have investigated to what extent the current automatic support provides useful help in accessibility validation activities, and what requirements a novel monitoring platform should provide to be a valid support in managing accessibility over time with respect to the accessibility standards and the WAD directive. For this purpose, we have carried out various activities aiming at better understanding the problems experienced by relevant user groups (developers, designers, experts and policy makers), and their accessibility knowledge and current work processes in order to identify user requirements for developing and providing a sustainable framework for the periodic monitoring and assessment of the Web accessibility state. This paper summarizes and discusses such activities useful for identifying requirements for large scale Web Accessibility evaluation.

## State of the Art in Guidelines and Automatic Tools for Web Accessibility Evaluation

The recent European legislation has stressed the importance of the right of people with disabilities to have access to online information and services of the public sector bodies. In fact, the European Accessibility Directive (WAD) 2016/2102 requires websites and Web applications to be compliant to the Web accessibility guidelines and standards, namely the European standard EN 301 549, which refers currently to the Web Content Accessibility Guidelines (WCAG) 2.1. In addition to this, it requires that public sector bodies periodically monitor the accessibility level of the Web services and compile a Web Accessibility Statement regarding their monitored Web services.

In this perspective automatic evaluation of Web accessibility [1] can play a useful role. Thus, we carried out a first analysis of the currently available solutions in terms of tools, which can support stakeholders in assessing accessibility compliance, detecting barriers, supporting Web developers in knowing how to solve them and commissioners and public bodies in monitoring the accessibility over time.

Such analysis indicated that support for the latest version (2.1) of the WCAG, which is the W3C recommendation since June 2018, is still limited because of the difficulties for several accessibility tool developers to easily update the functionalities of their validators. One possible tool architecture that supports the update of the guidelines to validate with limited effort is described in [9]. Moreover, among the tools that consider the recent guidelines, there are some that consider just a few features of Web pages (such as the colour contrast between foreground and background items), others that are not freely available, or that do not offer the checking against the AAA level of conformance. Often the analysis is limited to single pages and not to group of pages or even entire Web sites. As regards detecting barriers and showing them in the evaluation results, we noticed that the provided reports in some cases code-oriented, which means that issues of non-compliance are showed in the code line where they occur, thus it could be difficult for commissioners and experts who do not have Web programming knowledge to fully understand accessibility barriers in order to better act upon them.

Overall, a solution to Web accessibility assessment, comprehensive of all the possible useful features is still lacking. Investigations of accessibility evaluation tools conducted in the past have actually pointed out various issues [2, 4, 6–8]. They already revealed that such tools should provide more support for developers in conducting accessibility audits as well as in understanding and fixing errors. It would be desirable for interested users that a validation environment provides customized presentations of its results based on their role. The majority of the tools do not provide the visualization of the errors/warnings on the rendered Web page. WAVE was an exception since it provides this kind of report, while also providing the pinpointing of problems in the source code. Regarding commercial solutions, the Deque’s aXe browser extension and Siteimprove extension allow the developer to highlight issues on the running Web page. In general, there are accessibility-related features in which Web developers are more interested, and others that are more relevant to non-accessibility experts and public officials. In the end, only a few of the analyzed tools provide, in the full report, a dashboard that keeps track of the accessibility improvements, allowing the monitoring of the accessibility status over time. Such advanced feature is mainly provided by commercial services and solutions, whereas freely available tools and plugins provide users with an evaluation of the current version of the Website or Web page, without keeping track of previous accessibility evaluation results and improvements over time. Beyond this, assessment results are in the majority of cases reported and persisted using proprietary formats, i.e. several tools do not follow the Evaluation and Report Language (EARL) recommended by W3C^2^. The imergo® Web Compliance Suite has used EARL as assessment result format from the beginning in its developments. This decision was as well followed in the design of the WADcher data model, so all assessment results are conforming to EARL and are serialized as JSON-LD objects. One further issue is that the validation tools need to be able to provide their results in different formats that consider the different stakeholders that can use them, as it happens in MAUVE++  [3].

## Investigating Knowledge Level About Accessibility Evaluations and Resulting User Needs

In the following, we present the results of elicitation activities that we carried out in WADcher in order to collect experiences and feedback from Web commissioners, developers and content authors of Websites and Web applications. A similar research work regarding the extent to which the importance of Web accessibility is perceived by potential users, and how accessibility is actually implemented was done [5] some years ago; but accessibility standards have evolved as well as the Web technologies, thus the results of our work can provide an updated overview on such topic.

### Methodology

The project’s objective is to develop a supportive environment that helps the aforementioned WADcher target groups to create and maintain over time accessible products, thus we need to understand if they already use tools to exploit automatic support in ensuring the accessibility of the Web resources, and what are the functionalities they would like to exploit in order to better manage the accessibility evaluation and monitoring.

We carried out three main requirements elicitation activities: online questionnaires, interviews and a workshop. The objectives of our user research were to understand:

- to what extent people working in Web content development and commissioning are considering accessibility validation in their working routine;
- what is the perceived importance of Web accessibility compliance in the organizations they work; what their level of knowledge regarding Web accessibility standards is;
- what kind of accessibility evaluation activities they perform (manual, automatic, evaluation with experts);
- if they take advantage of using automatic evaluation tools and plugins.

In addition to these objectives, we wanted to investigate what information stakeholders look for in the reports generated by automatic accessibility evaluation tools; what kind of information and support they need from such tools, and what the features that they do not find but they would like to have available in them are.

We designed two multi-language online questionnaires to be filled remotely, one addressed to Web commissioners and people in charge of maintaining Websites and their accessibility, and one addressed to more technical stakeholders, namely Web developers and content authors. Questionnaires were divided into multiple sections, each one aimed to gather the following information: personal information and professional background; how they consider accessibility during work (for the commissioning or development part); their knowledge about Web accessibility guidelines, standards, and best practices; their knowledge regarding automatic accessibility assessment tools; suggestions based on what they like or dislike of such tools, and on what feature(s) they would like them to have to be a helpful support. The questionnaires were disseminated across the European countries from which partners of the WADcher project come from (Ireland, Italy, Greece, Austria, and Germany), through mailing lists of people employed in the public sector and personal contacts. Then, we interviewed some stakeholders. One interview was done in Germany and it involved a Web content author/accessibility expert; other two interviews were conducted in Italy and involved a Web content editor and a Web editorial staff member.

Moreover, a workshop was organized in Italy at AgID^3^ (AgID is the Italian organization in charge of monitoring the accessibility of public Web sites). The workshop was attended by about 40 people who work in accessibility in different organizations and with different roles. We involved them in a moderated discussion regarding accessibility standards and the WAD directive, their current workflow in considering accessibility, obstacles in considering and evaluating accessibility of the Web resources they are responsible of.

### Results

#### Online Survey.

We gathered feedback from 387 Web commissioners and from 148 Web developers and content authors through online questionnaires; people came from five European countries (Ireland, Italy, Greece, Austria, and Germany). As regards Web commissioners, 223 respondents are males and 164 females; they have an age ranging from 21 to 75 years old (mean 49; σ: 8, 7). They are mainly employees of organizations of medium size operating in the public sector (mainly at a local level: municipalities and schools). Most of them (131) have a management role (e.g. ICT/IT managers, school officials); 52 are responsible for websites; 44 have a technical role. As regards Web developers and content authors, 99 respondents are males and 49 females; their age is ranging from 25 to 72 years old (mean 44,4; σ: 10, 4). They are mainly employees of large companies and organizations, with a medium or high level of expertise in Web accessibility.

A first general consideration is that people involved in our elicitation activities have limited knowledge of accessibility assessment tools, and they usually encounter difficulties in both considering accessibility and evaluating it in their projects.

Web commissioners who answered the online questionnaire for the great majority have an intermediate level of accessibility knowledge, even if the same people answered that they have an elementary level in knowing the problems faced by people with disabilities in accessing the Web. The accessibility topic is perceived moderately important in their organizations: this can be due to the fact that they do not have a proper education in this topic, or that the accessibility compliance is not a requirement for their websites. But for commissioners who consider accessibility as a requirement there are difficulties in managing it in their workflow, and the reasons of such difficulties are the limited knowledge regarding standards (the 28,4% of them does not know any accessibility guideline and standard) and how to make Web resources compliant, and lack of time and resources. In terms of accessibility evaluation methods used they indicated: automatic evaluation with tools (148), manual guidelines review (142), HTML/CSS validation (127), expert testing (73), test with users (71), inspection with assistive technologies (64).

The answers from Web developers and accessibility experts, instead, indicate largely good knowledge regarding accessibility; only the 16,2% does not know any accessibility guideline and standard. In their workflow, they usually consider accessibility compliance in the final development phase. In fact, in most experiences, automatic evaluation tools are used in the pre-release step, when the product is almost ready. We noted that a lot of respondents from Italy are working in the educational sector. Web developers and content authors who work in this field, but in general those who work for public administrations, have to do with Web applications addressed to citizens of all ages who have the right to be informed and benefit from the public services provided via Web: it is of crucial importance that the contents be accessible to everyone, included people who need to access Web services using assistive technologies. In general, when they cannot work towards ensuring that Web applications meet the accessibility standards is because of commissioner’s imposition, lack of time and knowledge, and budget limitations.

#### Workshop.

The workshop had a broad audience: 19 Web developers, 11 content providers, 9 accessibility experts and evaluators, 1 blind person who participates to evaluation tests. They are employed in the fields of health care, government, education, banking, PAs (mainly large organizations). In the workshop some organizations reported that the accessibility checks of their websites are performed with tests with impaired users, HTML and CSS validation, and manual evaluation by accessibility experts. There is still a lack of culture about accessibility in the organizations where they work, but it emerged that organizations are more aware about accessibility if disabled people work within them. During the discussion, developers mentioned as one of the problems they face in considering accessibility is to maintain it over time and not just at the first release of the application. Examples of expected features in tools for accessibility evaluation that emerged are: the possibility to have customization options, such as filter relevant information by disability type, and/or by interface element type; a hierarchical analysis of the pages that compose the Website (in particular, the analysis of pages that are more subject to uploads and editing); the possibility to store the log of the identified errors, in order to be able to do the comparison the before and after situation, in a monitoring perspective; contextual help: help contextual to errors/alerts with links to checkpoints and additional examples, extraction of results with type of error (perhaps a code), URL, and location of the problematic element, in order to use results in next scripts; analysis of pages based on the device (to avoid that hidden elements, such as menus in mobile version, are analyzed), the analysis of PDF documents’ accessibility.

#### Interviews.

From the interviews carried out we gathered suggestions that emerged also from the questionnaires’ answers, such as: integrating accessibility guidelines into Content Management Systems used to update the websites, allowing the sorting of results by elements/topics (for example, tables, images, operability by keyboard), providing links and tips for possible solutions of the identified issues of non-compliance.

#### Discussion.

Unfortunately, not all commissioners and developers from whom we gathered feedback had used accessibility evaluation tools; in particular, 55% of the commissioners and 31% of the developers had not used them at all.

We asked people who used them at least once if there are obstacles in using them that they want to point out. As regards the support given by automatic evaluation tools, sometimes developers find difficulties in including dynamic content in the evaluation process, in the restrictions imposed by Content Management Systems, but also in other limitations of such tools, such as the detection of several false positives; the limited guidance on how to fix the detected issues; the effort they make in understanding the explanation of the violated success criterion and in general of the detected issue, thus, for example, they would like to have also suggestions on how to solve them.

Based on their knowledge of available accessibility assessment tools, they recommended the features they would expect from them. Among those, in general, they indicated a report tailored to the technical level of the user, by providing one more technical report addressed to developers, and one addressed to commissioners and content authors; the support in checking also dynamic content; giving measures representing the overall accessibility level reached.

Web commissioners, in particular, stressed the importance of having access to a non-technical report, where issues and trends are showed in a more graphical manner, without the details of the code, or in general the adaptation of the results report to the user role. They would appreciate that issues are grouped by gravity, that is by the impact level on the overall accessibility, and by the element(s) that are affected by the reported violations, so to organize the interventions in the code by groups of elements. They are also interested in knowing which categories of disabled users are more affected by the issues, and, in relation to this, they mentioned that it could be useful to filter the results by type of disability.

Web developers expressed the need to have more supporting information in relation to the identified issues: more and clearer examples of the identified violations, tips for the solution of the error, examples of interventions in the code in order to solve the barrier, customized solutions, showed in tooltips or modal sections. Then, they would like the possibility to aggregate recurring errors and to filter the potential false positives. In the end, they need that tools are able to efficiently evaluate also the dynamic code (analysis of scripts, analysis in the various states of the application). Another feature they would appreciate to better manage the fixing of the detected issue is the note-taking functionality, or being able to compile a to-do list of the interventions. The possibility to store the log of the identified errors, in order to be able to compare the before and after situation, in a monitoring perspective is also expected from automatic tools. They are also interested in metrics, such as the frequency of each error type (based, for example, on the different success criteria), a percentage representing how much the evaluated resource is still not accessible, the compliance level.

## Conclusions

The recent European directive requires structured monitoring of existing public Web applications, which can be achieved only with automatic support. The results gathered in the presented study provide useful insights on how stakeholders address issues concerning accessibility evaluation, know and use methods and tools for accessibility evaluation, and what they need for efficient evaluation and monitoring of the Web resources of their interest. In addition, they also provide indications of how accessibility is perceived in organizations and the level of importance it has. Overall, this study has made it possible to better understand to what extent accessibility assessment tools are used and provide useful support, and how they can be improved. More detail on the elicitation activities carried out is available in the project deliverables.

The insights gathered from both the analysis of the available solutions and the elicitation of users’ needs can be useful for all those who design and develop automatic support for accessibility evaluation, and have been used as a basis for the initial design of the WADcher tools. In fact, WADcher is intended as a set of tools composing a platform where accessibility evaluation results are accessed by Web commissioners, accessibility experts, Web developers and the monitoring bodies of the European countries. Each of these stakeholders represents a user role with specific requirements that emerged during our research and which have been considered in the design phase. In particular, the WADcher platform includes two main environments: a) the Observatory, which provides overview information on audits and trends over time of the evaluated sites, a dedicated area for compiling the Web Accessibility Statement as well as services targeted at national monitoring bodies and b) the Decision Support Environment, which for each audit supports the use of the results provided by external automatic evaluation tools to fix reported errors, and resolve audit results where expert decision is needed. The first one is addressed to the needs of Web commissioners and the monitoring bodies; the second one, instead, is addressed to developers and accessibility experts.

## Footnotes

1 https://wadcher.eu/.

2 https://www.w3.org/WAI/standards-guidelines/earl/.

3 https://www.agid.gov.it/.
